# Detection of Microvascular Failure After Thrombectomy Directly in the Angio-Suite Using Parametric Color Coding

**DOI:** 10.1007/s00062-025-01557-w

**Published:** 2025-08-19

**Authors:** Ludwig Singer, Maximilian Sprügel, Jeannette Becker, Hannes Lücking, Stefan T. Gerner, Stefan Schwab, Arnd Dörfler, Tobias Engelhorn

**Affiliations:** 1 Department of Neuroradiology, Friedrich-Alexander-Universität (FAU) Erlangen-Nürnberg, Schwabachanlage 6, 91054 Erlangen, Germany; 2 Department of Neurology, Friedrich-Alexander-Universität (FAU) Erlangen-Nürnberg, Schwabachanlage 6, 91054 Erlangen, Germany

**Keywords:** Acute ischemic stroke, No-reflow phenomenon, Intra-arterial thrombolysis, Parametric color coding, Microcirculation

## Abstract

**Purpose:**

The “no-reflow phenomenon” refers to persisting microvascular failure despite complete macrovascular reperfusion. We investigated whether parametric color coding (PCC) analysis of the DSA-series after successful mechanical thrombectomy could predict microvascular dysfunction.

**Methods:**

We retrospectively analyzed the STAMINA database for patients admitted to a single tertiary care center over a 5-year period with MCA-Occlusion (M1 or M2 branch), large penumbra, TICI 3 and infarct volume exceeding 15 ml on follow-up CT—presumed to reflect microvascular failure. These 55 patients were compared to 55 controls with infarcts < 15 ml on follow-up CT. As proof of concept, we included 42 non-stroke patients undergoing elective treatment of unruptured intracranial aneurysms. Using iFlow-PCC software, we calculated critical flow parameters, including cerebral circulation time (CirT), relative cortical time to peak (rTTP), and microvascular transit time (mTT).

**Results:**

Microvascular transit time (mTT) was significantly prolonged in the suspected microvascular failure group (3.22 ± 0.85 s) compared to the recanalization control group (2.79 ± 0.64 s; *p* = 0.003) and the non-stroke interventional control group (2.54 ± 0.90 s, *p* = 0.0003). The group with suspected microvascular failure exhibited a median modified Rankin Score at 3 months (mRS 3M: 4, IQR: 3–5) and a higher number of poor outcomes (mRS 5–6; *n* = 16) compared to the control group with similar strokes (median mRS 3M: 2, IQR: 1–4; mRS 5–6: *n* = 4).

**Conclusion:**

Prolonged microvascular transit time (mTT) can predict microvascular failure after thrombectomy. Angiographic flow analysis, performed directly in the angio-suite allows early identification of patients who may benefit from additional therapy like intra-arterial thrombolysis.

**Supplementary Information:**

The online version of this article (10.1007/s00062-025-01557-w) contains supplementary material, which is available to authorized users.

## Introduction

Endovascular therapy (EVT) for large vessel occlusion (LVO) in acute ischemic stroke has been established as standard of care for selected patients [1–6]. Advances in technology and methodology have improved recanalization rates above 80% [7, 8]. However, despite successful recanalization of the large vessel occlusion (LVO) a substantial subgroup of patients does not reach full functional independence defined as a modified Rankin Score (mRS) of 0–2 at 90 days after stroke [7, 9]. Several factors such as procedure time or the number of recanalization passes have been identified as predictors of poor neurological outcome [10, 11]. Additionally, the failure of microvascular recanalization despite sufficient macrovascular recanalization was shown to be a reason for large infarct size and subsequent poor neurological outcome. This phenomenon was termed “No-Reflow Phenomenon” [12, 13]. Its prevalence varies widely across studies but can be assumed to be roughly one in three patients [14]. Due to the multifactorial nature of outcome after mechanical thrombectomy, the no-reflow phenomenon is inherently difficult to diagnose. However, its early detection may offer an opportunity to initiate adjunctive therapies counteracting microvascular failure.

To find early imaging markers to predict the occurrence of no-reflow phenomenon we conducted a retrospective analysis of the Stroke Research Consortium in Northern Bavaria (STAMINA) database trying to identify an imaging marker for the “no-reflow”-phenomenon using parametric color coding (PCC) flow analysis of the post-recanalization digital subtraction angiography (DSA) series [15–17]. This software overlays each image of the DSA-series into a single, color-coded image allowing temporal quantification of blood flow. Through this it is possible to calculate several important hemodynamic parameters like cerebral circulation time (CirT) but also relative cortical time to peak (rTTP) and microvascular transit time (mTT) [18]. We hypothesize that quantification of cerebral hemodynamics within the angio-suite enables identification of patients at risk for microvascular reperfusion failure, potentially enabling stratification for adjunctive intra-arterial therapies, including thrombolytic or vasodilatory agents such as calcium channel antagonists [19, 20].

## Methods

The STAMINA (Stroke Research Consortium in Northern Bavaria) database is comprised of more than 5000 patients that were treated for acute ischemic stroke at the University Hospital of Erlangen since 2006. A retrospective data search of five consecutive years identified 270 patients with occlusions of the middle cerebral artery (MCA) segments M1 and M2. Further inclusion criteria were an initial ASPECTS Score ≥ 6, large Penumbra, Thrombolysis in Cerebral Infarction score (TICI) of 3 and an infarction volume on the 24 h-control CT of more than 15 ml resulting in 89 Patients. From these, 55 (men: 24, women: 31; age 76 IQR: 68–80) had adequate angiographic imaging for post-processing analysis. An additional 55 patients (men: 23, women 32; age: 74 IQR: 66–83) that underwent thrombectomy in the same timeframe in our institution but had an infarction volume of less than 15 ml on the follow-up CT were selected as our control group. We used a 15 ml infarction volume cut-off on follow-up CT, as this volume approximates the basal ganglia, which often show infarctions after MCA occlusion due to lack of collateral circulation [21, 22]. Consequently, the basal ganglia do not play a significant role in our task of assessing microvascular failure.

Patients that (i) developed vasospasms, (ii) had a relevant stenosis of the internal carotid artery, (iii) suffered from recurrent stroke during hospital stay or (iv) had an intracranial bleeding post-thrombectomy were excluded as these factors may contribute to confounding bias.

Additionally, as a proof of concept we analyzed 42 (men: 21; women: 21; age 61 IQR: 54–69) patients that were treated for unruptured intracranial aneurysms of the anterior circulation in our institution. This non-stroke control group underwent endovascular treatment of unruptured aneurysms that had not previously been treated to avoid the influence of hemodynamic effects of e.g. intracranial stents or flowdiverters.

Groups were selected to ensure comparable physiological conditions, as all patients were sedated and/or intubated during procedures. DSA series were performed with similar position of the 6‑French long sheath or guide catheter across all groups to allow for consistency in contrast media passage. The non-stroke control group was included due to the absence of a large vessel occlusion, but with similar physiological conditions. This consistency in image acquisition allows for a reliable comparison of cerebral flow patterns between groups with and without large vessel occlusion.

### Image Acquisition

The DSA was performed on a monoplane or biplane flat-panel detector angiography system (Axiom Artis dBA or Axiom Artis zeego, Siemens Healthineers, Forchheim, Germany). In all patients a standard transfemoral approach was used. Image acquisition was performed using a 6-French long sheath or guide catheter positioned in the cervical or subpetrous portion of the ICA. The 2D DSA series were acquired with a rate of 4 frames per second, as is routine in our department. For image acquisition, 8 ml of diluted contrast material (Imeron 300, Bracco Imaging, Konstanz, Germany) was injected in all series manually by an experienced operator at a flow rate of approximately 4 ml/s for 2 s validated by Gölitz et al. [18].

### Postprocessing (Parametric Color Coding)

Angiographic data was transferred to a Leonardo workstation (Siemens Healthineers, Forchheim) and evaluated using commercially available iFlow software (syngo iFlow, Siemens Healthineers, Forchheim) for evaluation. Each image of the 2D DSA image is overlayed with each other and the time to maximum opacification is color-coded. Additionally, in freely chosen points of interest (POI) and regions of interest (ROI), the precise time to peak opacification (TTP) can be calculated and displayed.

### Image and Statistical Analysis

Flow analysis was performed on the posterior-anterior (PA) and lateral (LAT) image of the post recanalization DSA series. In total 4 POIs were placed, 3 in the distal internal carotid artery (infracavernous, cavernous and supraophthalmic) and 1 in the superior sagittal sinus about 2 cm above the confluens of sinuses. Additionally, we placed 3 ROIs with a size of about 720 mm^2^ to display the cortical TTP replicating the apical ASPECTS-Regions of the MCA (M4–M6). We limited our dataset to patients with MCA occlusions at the level of M1 and M2-segments as the supplied brain parenchyma can be evaluated best on the lateral DSA series.

**Fig. 1:**
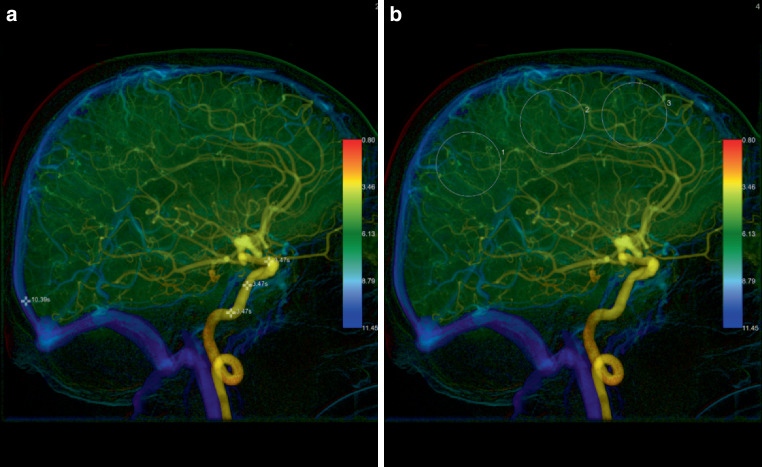
Color-coded image from a lateral 2D DSA series of a 63-year-old patient shows arterial POI placement at the carotid artery and venous POI placement at the superior sagittal sinus, positioned 2 cm anterior to the confluence of sinuses. The microvascular transit time (*mTT*) is assessed by calculating the time interval between the average TTP across all regions of interest (*ROIs*) compared to the TTP at the sagittal sinus. In this patient, who subsequently developed a large infarction the microvascular transit time (*mTT*) was calculated to be 4.78 s

**Fig. 2:**
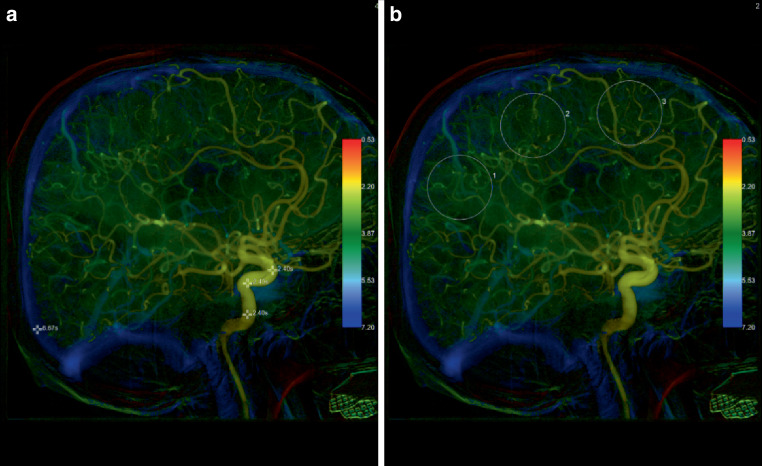
A color-coded image from a lateral 2D DSA series of a 75-year-old patient. Analogous to Fig. 1 POIs and ROIs were placed. This patient was discharged from hospital with an NIHSS score of 2 with only a minor infarction on subsequent CT imaging. The microvascular transit time (*mTT*) was calculated to be 2.40 s

We adapted the method of Greitz et al. and Gölitz et al. to define several important parameters of cerebral circulation. The cerebral circulation time (CirT) as the time difference from carotid siphon to the superior sagittal sinus [18, 23]. Depending on catheter position the maximum concentration of the carotid siphon was defined as the average between the POIs placed in the infracavernous and supraophthalmic regions of the ICA. For each of the cortical ROIs placed the relative cortical time to peak (rTTP) was calculated as the time difference between the carotid siphon and the TTP of each ROI placed. The microvascular transit time (mTT) was defined as the difference between TTP of a cortical ROI and the superior sagittal sinus. Readers were blinded during evaluation and POI/ROI placement to the outcome of patients to reduce potential bias. Exemplary results are shown in Figs. 1 and 2.

To identify factors independently associated with a favorable 90 days after stroke defined as mRS ≤ 2, we fitted a multivariable logistic regression with mRS ≤ 2 as the dependent variable. Furthermore we explored which clinical and procedural variables influence microvascular transit time and performed an ordinary least-squares linear regression using mTT as the outcome; regression coefficients are presented with 95% confidence intervals.

Statistical analysis and graphical representation was performed using python and R [24, 25]. Mean values and standard deviation (SD) was calculated for CirT, cortical rTTP and mTT. Group differences were tested for normality using the Shapiro-Wilk test and compared using the Mann-Whitney U test or independent samples t‑test, as appropriate. For functional outcomes, the modified Rankin Scale (mRS) was dichotomized at 0–2 (favorable) vs. 3–6 (unfavorable), and group differences were assessed using Fisher’s Exact Test. A *p*-value ≤ 0.05 was considered statistically significant.

## Results

### Patient Characteristics

We evaluated 55 patients (men: 24; women: 31; age 73.2; IQR: 68–80) with M1/M2 occlusion and an infarction volume on follow up CT of more than 15 ml. For our control group we selected 55 patients (men: 23, women 32; age: 74; IQR: 66–83), which met the same criteria but with an infarction volume on follow-up CT of less than 15 ml. Further inclusion criteria followed the guidelines of the European Stroke Organization (ESO) on endovascular therapy for acute ischemic stroke with an ASPECTS ≥ 6 and time window < 24 h [26].

The median time interval between stroke onset and hospital arrival in the group with suspected microvascular failure was 311 min (IQR: 23–1222 min) compared to patients without suspected microvascular failure (215 min; IQR: 27–931) at no statistical relevance (*p* = 0.3255). The average ASPECTS score at the primary stroke center was 8 (IQR: 7–10) for those with suspected microvascular failure and an ASPECTS of 9 (IQR: 8–10) in the control (*p* = 0.049).

As a proof of concept, we included a second control group of 42 patients (21 male, 21 female; median age 61.3 years, IQR 54–69) who underwent elective endovascular treatment of unruptured anterior circulation aneurysms, chosen for their comparable procedural conditions and catheter positioning during injection. We decided against including more patients into this group as there was little variance in the measured cerebral circulation parameters and relevant statistical significance was reached.

### Procedural and Angiographic Flow Analysis

**Fig. 3:**
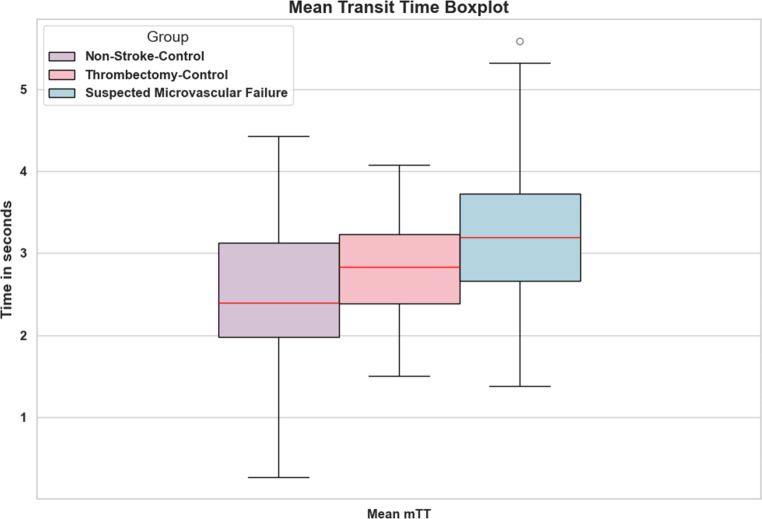
Boxplots of microvascular transit times (*mTT*) of the groups analyzed. Patients in the group with suspected microvascular failure exhibit significantly prolonged microvascular transit times (*mTT*), compared to the control groups

The suspected no-reflow group showed significantly prolonged mTT (3.22 s ± 0.85) compared to both the recanalization control (2.79 s ± 0.65, *p* = 0.003) and the interventional control group (2.54 s ± 0.90, *p* = 0.0003). Cerebral circulation time showed no statistically significant difference between the group of suspected microvascular failure (5.92 s ± 1.26), the recanalization control group (5.65 s ± 1.15), and the interventional control group (5.80 s ± 1.30) with *p* = 0.54 vs *p* = 0.24 respectively. Mean relative cortical time to peak in the suspected no-reflow group (2.71 s ± 0.99) compared to the recanalization control group (2.86 s ± 0.99) while the interventional control group (3.26 s ± 0.90) showing statistical significance between the non-stroke control group and the suspected microvascular failure group (*p* = 0.0048) (Tables 1 and 2; Fig. 3). The median amount of recanalization attempts (passes) in the group with suspected microvascular failure was 2 (IQR: 1–3) compared to the control group of 2 (IQR: 1–2). Sensitivity analyses regarding microvascular transit time (mTT) yielded similar results. After adjustment for ASPECTS, age, initial NIHSS, interval between stroke onset and hospital arrival and number of thrombectomy passes, patients in the suspected no-reflow group showed significantly prolonged mTT compared to controls (β = 0.35 s, SE = 0.15; *p* = 0.024).

**Table 1:** Summary of critical hemodynamic flow parameters. Means ± standard deviation for Microvascular Transit Time, Cerebral Circulation Time, and Relative Cortical Time to Peak in the Suspected No-Reflow, Recanalization Control, and Interventional Control groups.

	Mean Microvascular Transit Time (s)	Mean Cerebral Circulation Time (s)	Mean Relative Cortical Time to Peak (s)
Suspected No-Reflow (*n* = 55)	3.22 ± 0.85	5.92 ± 1.26	2.71 ± 0.99
Recanalization Control (*n* = 55)	2.79 ± 0.64	5.65 ± 1.15	2.86 ± 0.99
Interventional Control (*n* = 42)	2.54 ± 0.90	5.81 ± 1.31	2.71 ± 0.99

**Table 2:** Statistical comparison of hemodynamic parameters. *P*-values for pairwise comparisons between study groups for Microvascular Transit Time, Cerebral Circulation Time, and Relative Cortical Time to Peak.

	Mean Microvascular Transit Time (*p*-value)	Mean Cerebral Circulation Time (*p*-value)	Mean Relative Cortical Time to Peak (*p*-value)
Suspected No-Reflow vs Recanalization Control	*p* = 0.003	*p* = 0.2429	*p* = 0.0048
Suspected No-Reflow vs Interventional Control	*p* = 0.0003	*p* = 0.6704	*p* = 0.0376
Recanalization Control vs Interventional Control	*p* = 0.1309	*p* = 0.5382	*p* = 0.4289

### Clinical Outcomes

**Fig. 4:**
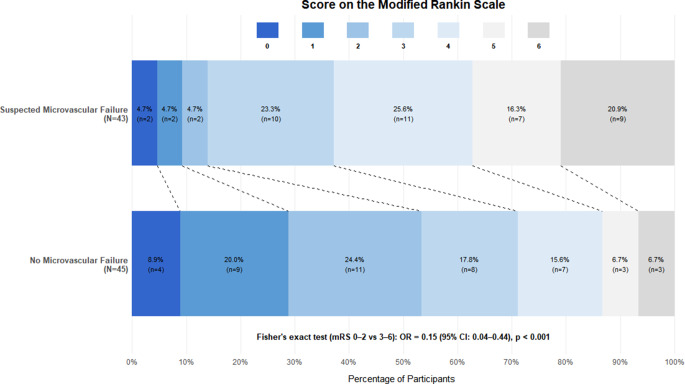
Clinical outcomes. A significantly larger proportion of patients achieved functional independence (mRS 0–2) at 3 months in the group without suspected microvascular failure (*n* = 15) compared to the group with suspected microvascular failure (*n* = 6). Fisher’s exact test confirmed that patients with suspected microvascular failure had significantly lower odds of achieving a favorable outcome (OR = 0.15, 95% CI: 0.04–0.44, *p* < 0.001), indicating a strong negative association

Median initial NIHSS score at presentation was 18 (IQR: 14–20) for the group with suspected microvascular failure versus 14 (IQR: 10–18) in the control group. (*p* = 0.0629). At discharge, the median NIHSS score was 10 (IQR: 6–15) in the group of suspected microvascular failure and 4 (IQR: 2–6) in the control group, respectively, (*p* = 0.0001). Median modified Rankin Score (mRS) before the onset of symptoms was 0 (IQR: 0–2) in the group of microvascular failure and 0 (IQR: 0–1) in the control. At 3 months, median mRS was 4 (IQR: 3–5) in the suspected microvascular failure group and 2 (IQR: 1–4) in the control group, (*p* = 0.004). Notably, the number of patients with mRS scores of 5–6 was higher in the suspected microvascular failure group (16 vs 4), while the rate of patients achieving functional independence (mRS 0–2) was significantly higher in the control group (6 vs 15; Fig. 4). Post hoc analysis comparing dichotomized outcomes (mRS 0–2 vs. 3–6) revealed significantly lower odds of favorable outcome in the suspected microvascular failure group in univariate analysis (OR = 0.15, 95% CI: 0.04–0.44; Fisher’s Exact Test, *p* < 0.001). After adjustment for ASPECTS, age, initial NIHSS, interval between stroke onset and hospital arrival, mean mTT and number of thrombectomy passes belonging to the suspected microvascular failure group remained independently associated with significantly lower odds of favorable outcome (adjusted OR = 0.10, 95% CI: 0.02–0.39; *p* = 0.001).

## Discussion

Despite complete macrovascular recanalization patients with LVO treated with EVT frequently do not achieve favorable outcomes defined as mRS ≤ 2, at 90 days after stroke [27–29]. Many factors have been implicated in this, such as time interval between stroke onset and hospital arrival, initial NIHSS score, ASPECTS, EVT-procedure time, number of passes until recanalization is achieved, TICI score, hemorrhagic transformation and many more [27, 29, 30].

Recently, another factor has moved again into the spotlight, the “no-reflow” phenomenon. It describes the microvascular failure after macrovascular recanalization with a prevalence of up to one in three patients [14]. Therefore, the identification of a potential imaging marker to identify patients at risk for the no-reflow phenomenon is of critical importance, as it can guide subsequent therapeutic interventions, such as intraarterial lysis [19].

The precise mechanism behind the no-reflow phenomenon is not fully understood. It has been linked to several factors such as distal embolization and microvascular contraction [31, 32]. Ultimately, all these factors lead to microvascular obstruction which has even been discovered by MRI studies [33]. This additive hypoperfusion may lead to infarction expansion despite successful macrovascular recanalization and ultimately poor neurological outcome. Additionally, the decreased blood flow also impairs resolution of edema, clearance of necrotic tissue and collateral angiogenesis impairing tissue repair [32].

To identify this persisting hypoperfusion, we performed flow analyses of the routinely acquired 2D DSA control series after macrovascular recanalization to see if parametric color coding (PCC) could serve as a potential marker for the occurrence of the no-reflow phenomenon. We defined hemodynamic markers of cerebral circulation which have already previously been established including cerebral circulation time, microvascular transit time (mTT) and cortical time to peak for each patient [18, 23].

Among the hemodynamic parameters analyzed, microvascular transit time (mTT) was significantly prolonged in patients with suspected no-reflow compared to both non-stroke controls and the patients without extensive infarction. This suggests that even though macrovascular recanalization has been achieved there is persisting microvascular obstruction contributing to impaired tissue reperfusion. In contrast, cerebral circulation time (CirT) showed no significant differences between groups, indicating that it is a broader measure of global perfusion and may lack sensitivity to capture the subtle microvascular impairment in the setting of the no-reflow phenomenon. Relative cortical time to peak (rTTP) did not differ significantly between the stroke subgroups, but both exhibited faster cortical perfusion compared to controls—likely reflecting a hyperemic response commonly seen following reperfusion [34].

Our findings suggest that the microvascular transit time (mTT) measured by PCC could serve as a quick and robust marker of microvascular failure that can be obtained directly in the Angio-Suite after macrovascular recanalization. Another advantage of using the post-recanalization DSA series is that these images are routinely acquired after mechanical thrombectomy without requiring additional radiation exposure and contrast application.

Our findings align with other studies highlighting that flow analysis is a valuable tool in the measurement of cerebral flow parameters. Ji et al. demonstrated that microcirculation time after thrombectomy is a predictive marker for 90-day functional prognosis after stroke onset [35]. Importantly, in their study patients with a TICI ≥ 2 b were included. A key distinction of our analysis is that we included exclusively patients that had complete macrovascular recanalization, defined as TICI 3. This suggests that the prolonged microvascular transit time (mTT) in our cohort is not solely dependent on the degree of macrovascular recanalization but rather visualizes true microvascular failure.

We acknowledge that final infarct size is influenced by multiple variables beyond microvascular failure, including time to recanalization, occlusion localization, number of thrombectomy passes, collateral status and pre-existing comorbidities [36, 37]. While our study focused specifically on patients with complete macrovascular recanalization (TICI 3) to isolate the effect of microvascular dysfunction, we recognize that there are many confounding factors that may still influence outcomes.

Additionally, PCC-derived mTT should be correlated with pre-interventional CT/MR perfusion parameters to better understand the relationship between initial tissue status and post-recanalization microvascular dysfunction. Furthermore, correlation with flat panel perfusion CT performed directly in the angio-suite could support or even provide immediate validation of prolonged mTT findings and help distinguish areas of reversible hypoperfusion [38].

Overall, our findings further underline the potential of PCC allowing ultra early identification of possible microvascular failure directly in the Angio-suite. Importantly, PCC assessment is quick and easy to perform, can be conducted immediately after successful macrovascular recanalization, and provide insight into cerebral hemodynamics that can trigger additional therapies. Multicenter studies enrolling a larger cohort are essential to validate these findings and establish optimal PCC-derived mTT cutoff values.

Furthermore, prospective multicenter randomized controlled trials should evaluate potential therapeutic thresholds and assess patient outcomes when additional interventions—such as intra-arterial thrombolysis or calcium-channel antagonists—are implemented based on PCC guidance [19, 20].

### Limitations

While our findings underline the utility of microvascular transit time (mTT) as an early marker of the no-reflow phenomenon, our study is certainly limited by the small and heterogenous sample size, meaning that our findings can be considered only preliminary. Furthermore, the size of infarction on follow-up imaging is multifactorial and not only related to the no-reflow phenomenon. Although median time from symptom onset to hospital arrival and baseline ASPECTS differed slightly between our thrombectomy groups—and thus represent potential confounders, both variables were included as covariates in our multivariable models to adjust for their effects. Manual contrast injection, although validated in studies before, and the relatively low frame rate are a possible drawback in our dataset and in the future automatic injections and higher frame rates should be considered. These steps may improve image characteristics and allow more precise flow analysis at the cost of higher radiation exposure of the patient.

## Conclusion

Microvascular angiographic flow analysis can be used as an ultra-early marker for the occurrence of microvascular failure. Our results indicate that patients with successful macrovascular recanalization, but subsequent large infarction observed on follow-up CT, have a significantly prolonged microvascular transit time (mTT).

PCC-derived measurement of mTT provides a quick and easy method that can be performed immediately after recanalization in the angio-suite without the need for additional radiation exposure or contrast media, thus enabling rapid stratification of patients for adjunctive interventions.

However, prospective validation in larger, multicenter cohorts is needed to determine optimal mTT threshold to predict no-reflow and to assess whether PCC-guided therapies—such as intra-arterial thrombolysis or calcium-channel antagonists—improve clinical outcomes.

## Supplementary Information

**Online Resource 1:** The initial data size was 1407 patients in the STAMINA database treated for ischemic stroke from 2015–2020. 147 patients were with an M1 or M2 occlusion, TICI 3, ASPECTS ≤ 6. From the 147 patients, two subgroups were then analyzed, 89 patients with > 15 ml infarction on control CT and 90 patients with < 15 ml infarction on control CT. From these groups 55 and respectively 55 Patients had adequate imaging accessible for flow analysis using Siemens iFLOW PCC. The non-stroke control group was comprised of 23 patients undergoing elective treatment for unruptured intracranial aneurysms.

**Online Resource 2**: Detailed characteristics of the different Patient populations.

**Online Resource 3:** Boxplots of all critical cerebral flow parameters.

## Abbreviations

ASPECTS: Alberta stroke program early CT score
CirT: Cerebral circulation time
DSA: Digital subtraction angiography
EVT: Endovascular stroke therapy
ICA: Internal carotid artery
LVO: Large vessel occlusion
MCA: Middle cerebral artery
mRS: Modified Rankin Score
mTT: Microvascular transit time
NIHSS: National Institutes of Health Stroke Scale
NRP: No reflow phenomenon
PCC: Parametric color coding
POI: Point of interest
ROI: Region of interest
rTTP: Relative cortical time to peak
STAMINA: Stroke Research Consortium in Northern Bavaria
TICI score: Thrombolysis in Cerebral Infarction-score
