# Supplementary material for: Detection of Microvascular Failure After Thrombectomy Directly in the Angio-Suite Using Parametric Color Coding
Source: Clin Neuroradiol. 2025 Aug 19;36(1):67–75. doi: 10.1007/s00062-025-01557-w (PMC13009058; doi:10.1007/s00062-025-01557-w)
Supplement: Supplementary file 1 — Online Resource 1: The initial data size was 1407 patients in the STAMINA database treated for ischemic stroke from 2015–2020. 147 patients were with an M1 or M2 occlusion, TICI 3, ASPECTS ≤ 6. From the 147 patients, two subgroups were then analyzed, 89 patients with > 15 ml infarction on control CT and 90 patients with < 15 ml infarction on control CT. From these groups 55 and respectively 55 Patients had adequate imaging accessible for flow analysis using Siemens iFLOW PCC. The non-stroke control group was comprised of 23 patients undergoing elective treatment for unruptured intracranial aneurysms. [file 62_2025_1557_MOESM1_ESM.docx]

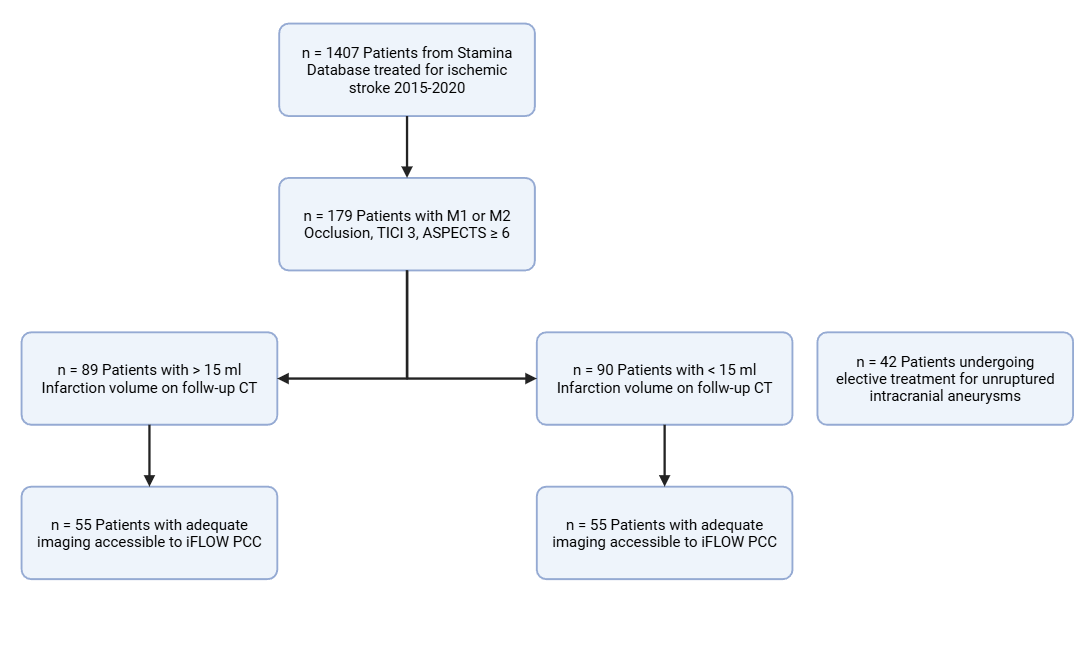


**Online Resource 1:** The initial data size was 1407 patients in the STAMINA database treated for ischemic stroke from 2015-2020. 147 patients were with an M1 or M2 occlusion, TICI 3, ASPECTS ≤6. From the 147 patients, two subgroups were then analyzed, 89 patients with >15ml infarction on control CT and 90 patients with <15ml infarction on control CT. From these groups 55 and respectively 55 Patients had adequate imaging accessible for flow analysis using Siemens iFLOW PCC. The non-stroke control group was comprised of 23 patients undergoing elective treatment for unruptured intracranial aneurysms.
