# Supplementary material for: Detection of Microvascular Failure After Thrombectomy Directly in the Angio-Suite Using Parametric Color Coding
Source: Clin Neuroradiol. 2025 Aug 19;36(1):67–75. doi: 10.1007/s00062-025-01557-w (PMC13009058; doi:10.1007/s00062-025-01557-w)
Supplement: Supplementary file 2 — Online Resource 2: Detailed characteristics of the different Patient populations. [file 62_2025_1557_MOESM2_ESM.docx]

| Characteristic | Interventional control group (N=42) | Suspected Microvascular Failure (N=55) | No Microvascular Failure (N=55) |
| --- | --- | --- | --- |
| Median age (IQR) — yr | 74 (65–78) | 76 (68–80) | 74 (66–83) |
| Male sex — no. (%) | 22 (40.0) | 24 (43.6) | 23 (41.8) |
| Median NIHSS score at admission (IQR) |  | 17 (14-20) | 14 (10-18) |
| Median NIHSS score at release (IQR) |  | 10 (6-15) | 4 (2-6) |
| Median modified Rankin Score before admission (IQR) |  | 0 (0-1) | 0 (0-2) |
| Median modified Rankin Score 3 months after stroke (IQR) |  | 4 (3-5) | 2 (1-4) |
| ASPECTS Value based on CT |  | 9 (7-10) | 9 (8-10) |
| Median interval between stroke onset and hospital arrival (IQR) — min |  | 200 (65-377) | 126 (70-249) |
| Number of CT-perfusion performed |  | 42 | 15 |
| Median Mismatch-volume on CTP |  | 87 (IQR 5-145) | 7 (IQR 2-49) |
| Number of passes |  | 2 (1-2) | 2 (1-4) |

Online Resource 2: Detailed characteristics of the different Patient populations.
