# Supplementary figures and images for: Detection of Microvascular Failure After Thrombectomy Directly in the Angio-Suite Using Parametric Color Coding
Source: Clin Neuroradiol. 2025 Aug 19;36(1):67–75. doi: 10.1007/s00062-025-01557-w (PMC13009058; doi:10.1007/s00062-025-01557-w)

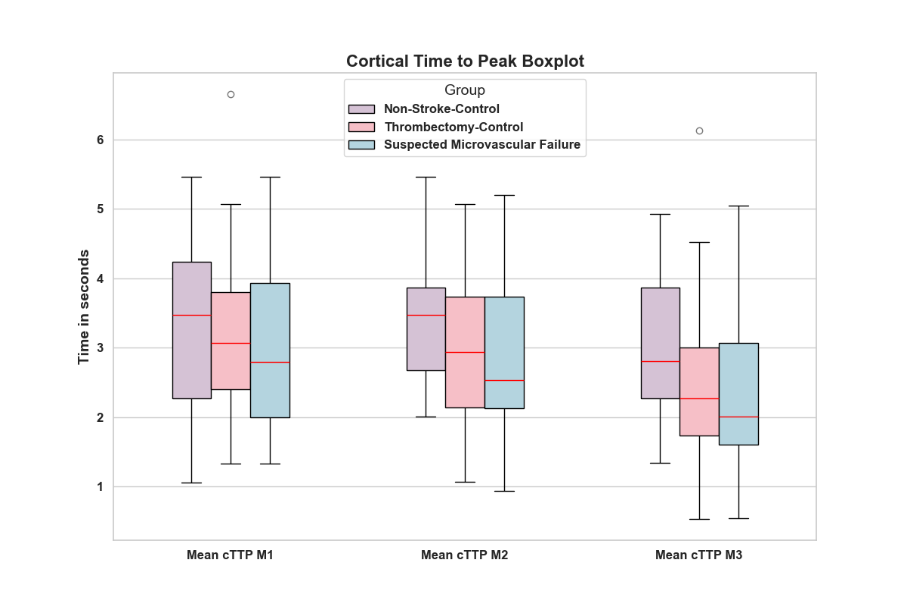

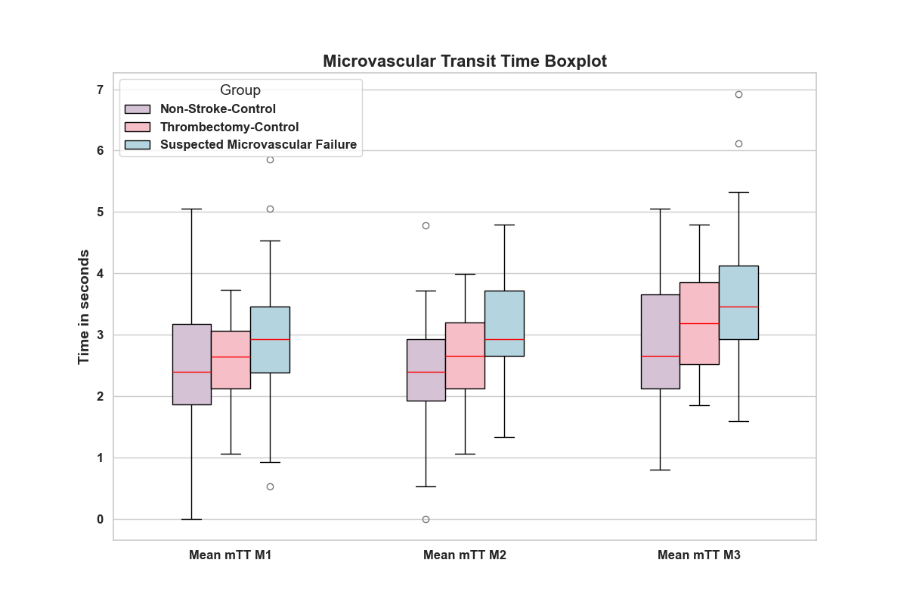

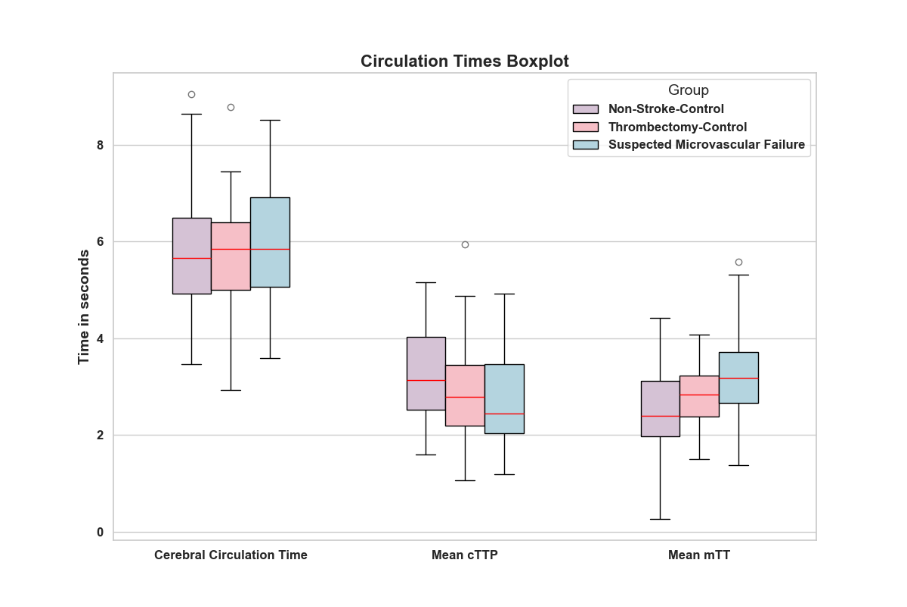


**Online Resource 3:** Boxplots of all critical cerebral flow parameters.

Supplement: Supplementary file 3 — Online Resource 3: Boxplots of all critical cerebral flow parameters. [file 62_2025_1557_MOESM3_ESM.docx]
